# An Updated Review of the Efficacy of Cupping Therapy

**DOI:** 10.1371/journal.pone.0031793

**Published:** 2012-02-28

**Authors:** Huijuan Cao, Xun Li, Jianping Liu

**Affiliations:** 1 Centre for Complementary Medicine Research, University of Western Sydney, Penrith, Australia; 2 Centre for Evidence-Based Chinese Medicine, Beijing University of Chinese Medicine, Beijing, China; Universidad Peruana Cayetano Heredia, Peru

## Abstract

**Background:**

Since 1950, traditional Chinese medicine (TCM) cupping therapy has been applied as a formal modality in hospitals throughout China and elsewhere in the world. Based on a previous systematic literature review of clinical studies on cupping therapy, this study presents a thorough review of randomized controlled trials (RCTs) to evaluate the therapeutic effect of cupping therapy.

**Method:**

Six databases were searched for articles published through 2010. RCTs on cupping therapy for various diseases were included. Studies on cupping therapy combined with other TCM treatments versus non-TCM therapies were excluded.

**Results:**

135 RCTs published from 1992 through 2010 were identified. The studies were generally of low methodological quality. Diseases for which cupping therapy was commonly applied were herpes zoster, facial paralysis (Bell palsy), cough and dyspnea, acne, lumbar disc herniation, and cervical spondylosis. Wet cupping was used in most trials, followed by retained cupping, moving cupping, and flash cupping. Meta-analysis showed cupping therapy combined with other TCM treatments was significantly superior to other treatments alone in increasing the number of cured patients with herpes zoster, facial paralysis, acne, and cervical spondylosis. No serious adverse effects were reported in the trials.

**Conclusions:**

Numerous RCTs on cupping therapy have been conducted and published during the past decades. This review showed that cupping has potential effect in the treatment of herpes zoster and other specific conditions. However, further rigorously designed trials on its use for other conditions are warranted.

## Introduction

Cupping is a traditional Chinese medicine (TCM) therapy dating back at least 2,000 years. Types of cupping include retained cupping, flash cupping, moving cupping, wet cupping, medicinal cupping, and needling cupping [1]. The actual cup can be made of materials such as bamboo, glass, or earthenware. The mechanism of cupping therapy is not clear, but some researchers suggest that placement of cups on selected acupoints on the skin produces hyperemia or hemostasis, which results in a therapeutic effect [2].

In our previous study, we conducted a systematic literature review based on available clinical studies published from 1958 through 2008 [3]. We concluded that the majority of the 550 included studies showed that cupping is of potential benefit for pain conditions, herpes zoster, and cough and dyspnea. Five other systematic reviews [4]–[8] on cupping therapy have also been published, focusing on pain conditions, stroke rehabilitation, hypertension, and herpes zoster, respectively. The numbers of included trials in these reviews were quite small (between 1and 8 trials). Lee et al. [9] conducted an overview of these five reviews and concluded that cupping is only effective as a treatment for pain, and even for this indication doubts remain. Extensive search did not find further related reviews.

Though the quality of included randomized controlled trials (RCTs) in the aforementioned reviews was generally poor according to the Cochrane risk of bias tool, we felt that it was still worth conducting an overview systematic review to further evaluate the therapeutic effect of cupping therapy for specific disease/conditions due to the paucity of evidence in this subject.

## Methods

The flow diagram for this review and supporting CONSORT checklist are available as supporting information; see Checklist S1 and Protocol S1.

### Inclusion Criteria

Eligible studies were randomized controlled trials (RCTs) that examined the therapeutic effect of cupping therapy, including one or more types of cupping methods, compared with no treatment, placebo, or conventional medication. Cupping combined with other interventions and compared with other interventions alone were also included. Studies that looked at cupping therapy combined with other TCM therapies, such as acupuncture, compared with non-TCM therapies were excluded. Multiple publications reporting the same patient data set were also excluded. There was no restriction on language and publication type.

### Identification and Selection of Studies

Based on our previous review [3], an updated search of publications was performed using China Network Knowledge Infrastructure (CNKI) (2009 through 2010), Chinese Scientific Journal Database (VIP) (2009 through 2010), Chinese Biomedical Database (CBM) (2009 through 2010), Wanfang Database (2009 through 2010), PubMed (1966 through 2010), and the Cochrane Central Register of Controlled Trials (CENTRAL, 1800 through 2010). All searches ended at December 2010. The search terms included *cupping therapy*, *bleeding cupping*, *wet cupping*, *dry cupping*, *flash cupping*, *herbal cupping*, *moving cupping*, *needling cupping* and *retained cupping*. Two authors (HC and XL) independently identified and checked each study against the inclusion criteria.

### Data Extraction and Quality Assessment

Two authors (HC and XL) independently extracted the data from the included trials. The extracted data included authors and title of study, year of publication, type of disease, study size, age and gender of participants, and methodological information. Other extracted data included type of cupping therapy, treatment process, control interventions, outcomes (for example, overall efficacy rate), and adverse effects.

Quality of included trials was evaluated. Methodological quality of RCTs was assessed using criteria from the *Cochrane Handbook for Systematic Reviews of Interventions* [10]. Trials were appraised according to the risk of bias for each important outcome, including adequacy of generation of the random allocation sequence, allocation concealment, blinding, and outcome reporting. Quality of each trial was categorized into low/unclear/high risk of bias. Trials that met all criteria were categorized into low risk of bias, trials that met none of the criteria were categorized into high risk of bias, and the remaining trials were categorized into unclear risk of bias if there was insufficient information to make a judgment.

### Data Analysis and Statistical Methods

Data were extracted using Microsoft Access and transferred into Microsoft Excel spreadsheets to be calculated for frequency. Outcome data were summarized using risk ratio (RR) with 95% confidence intervals (CI) for binary outcomes or mean difference (MD) with 95% CI for continuous outcomes. RevMan 5.0.20 software was used for data analyses. Meta-analysis was used if the trials had good homogeneity, which was assessed by examining *I^2^* (an index that describes the percentage of variation across studies that is due to heterogeneity rather than chance), on study design, participants, interventions, control, and outcome measures. Funnel plot analysis was done to determine publication bias.

## Results

### Basic Information of Studies

Searches of six databases identified 1,294 citations, the majority of which were deemed ineligible from reading title and abstract (Protocol S1). Full-text papers of 108 trials were retrieved. In addition to the 73 trials from our previous review, 62 new trials were included in this study. Of the 135 included trials [11]–[145], 132 were published in Chinese, including 3 unpublished dissertations [16], [27], [72].The remaining 3 trials [69], [73], [86] were published in English. All included studies were published from 1992 through 2010, with more than half from 2008 through 2010 (Table S1).

### Description of Interventions

**Figure 1:**
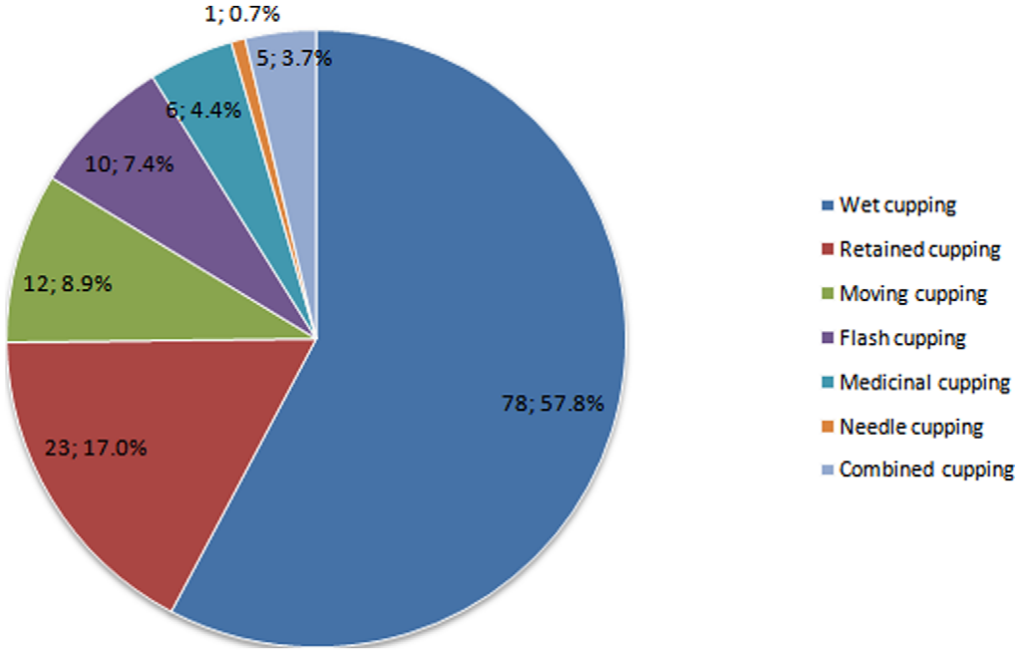
Constituent ratios of types of cupping therapy.

Among the included trials, 78 (57.78%) used wet cupping as the main intervention, 23 (17.04%) used retained cupping, 12 (8.89%) used moving cupping, 10 trials (7.40%) used flash cupping, 6 (4.44%) used medicinal cupping, and 1 (0.74%) used needle cupping. Combined cupping in which at least two types of cupping methods were applied, was used in 5 trials (3.70%) (Figure 1).

### Distribution of Diseases/Conditions

In the included trials, 56 diseases or symptoms were treated by cupping therapy. Diagnostic criteria varied, some authors used international criteria, such as ICD-10, others used Chinese criteria, such as those issued by government health agencies, or criteria from Chinese language medical textbooks. Some authors did not report any sources for their diagnostic criteria. The 6 most common diseases/conditions for which cupping was applied were herpes zoster (17 trials), facial paralysis (Bell palsy) (17 trials), cough and dyspnea (8 trials), acne (6 trials), lumbar disc herniation (6 trials) and cervical spondylosis (6 trials) (Table 1). Meta-analyses were conducted on 4 diseases/conditions – herpes zoster, facial paralysis (Bell palsy), acne and cervical spondylosis (characteristics of the RCTs involving these 4 diseases are presented in Tables S2, S3, S4 and S5). Due to the heterogeneity of the RCTs of the remaining 2 diseases/conditions – lumbar disc herniation and cough and dyspnea – meta-analyses could not be completed.

**Table 1:** Reporting of quality components in 135 included randomized clinical trials on cupping therapy.

Year published	No. of Randomized controlled trials	Adequate sequence generation (%)	Adequate allocation concealment (%)	Blinding method reported (%)	Incomplete outcome data (yes, %)	Selective outcome reporting (yes, %)	Comparability of baseline (yes, %)	Sample size estimation (yes, %)	Inclusive criteria (yes, %)	Exclusive criteria (yes, %)	Diagnostic standard (yes, %)
1992	2	0	0	0	0	0	0	0	0	0	0
1993	1	1(100%)	0	0	0	0	0	0	0	0	0
1994	1	0	0	0	0	0	0	0	0	0	0
1995	-	-	-	-	-	-	-	-	-	-	-
1996	-	-	-	-	-	-	-	-	-	-	-
1997	2	0	0	0	0	0	0	0	0	0	0
1998	1	0	0	0	0	0	0	0	0	0	0
1999	2	0	0	0	0	0	1(50%)	0	0	0	1(50%)
2000	2	0	0	0	0	0	1(50%)	0	1(50%)	2(100%)	2(100%)
2001	-	-	-	-	-	-	-	-	-	-	-
2002	-	-	-	-	-	-	-	-	-	-	-
2003	7	0	0	0	0	0	2(28.57%)	0	0	1(14.29%)	4(57.14%)
2004	8	0	0	0	0	0	6(75%)	0	3(37.5%)	1(12.5%)	3(37.5%)
2005	10	2(20%)	0	1(10%)	0	0	7(70%)	0	6(60%)	5(50%)	8(80%)
2006	19	5(26.32%)	0	1(5.26%)	1(5.26%)	0	14(73.68%)	1(5.26%)	4(21.05%)	4(21.05%)	15(78.95%)
2007	11	4(36.37%)	0	0	2(18.19%)	0	11(100%)	0	3(27.27%)	3(27.27%)	7(63.64%)
2008	12	5(41.67%)	0	1(8.33%)	0	0	10(83.33%)	0	4(33.33%)	4(33.33%)	10(83.33%)
2009	28	5(17.86%)	3(10.71%)	1(3.57%)	1(3.57%)	1(3.57%)	24(85.71%)	2(7.14%)	11(39.29%)	12(42.86%)	25(89.29%)
2010	29	3(10.34%)	0	0	1(3.45%)	0	25(86.21%)	0	9(31.03%)	7(24.14%)	25(86.21%)
**Total**	135	25(18.52%)	3(2.22%)	4(2.96%)	5(3.70%)	1(0.74%)	101(74.81%)	3(2.22%)	42(31.11%)	40(29.63%)	101(74.81%)

Of the 6 diseases/conditions, 3 were related to pain, including herpes zoster, an inflammatory pain of the nerve; and lumbar disc herniation and cervical spondylosis, pain caused by nerve compression. Relieving pain was the main purpose of cupping therapy in these studies. Retained cupping or wet cupping was typically applied.

Facial paralysis (Bell palsy) falls under nerve, nerve root, and plexus disorders. In the studies we reviewed, flash cupping and moving cupping were commonly applied.

Respiratory diseases, such as pneumonia, bronchitis, and asthma, for which the main purpose of treatment is to alleviate the symptoms of cough and dyspnea are also treated by cupping therapy. Retained cupping or wet cupping therapy on EX-B1, a so-called extra acupoint (acupuncture point not located on one of the traditional channels), was mostly used in the studies for treating cough and dyspnea symptoms.

Acne is a skin condition that affects the face, neck, shoulders, chest, and back. In the studies we evaluated, wet cupping was primarily used to relieve the skin breakouts.

The remaining 50 diseases/conditions are presented in Table S1.

### Methodological Quality of RCTs

According to our pre-defined methodological quality criteria, none of the 135 trials were low risk of bias and the majority was high risk of bias (Table 1). Three trials [23], [69], [73] reported sample size calculations, 25 trials [11], [14], [17], [23], [26], [32], [35], [41], [48], [57], [60], [69], [70], [72], [73], [79], [81], [85], [88], [94], [97], [99], [107], [111], [116] described randomization procedures (such as random number table or computer-generated random numbers), with only 2 [23], [73] of the 25 trials using sealed envelope allocation concealment. Four trials [17], [48], [94], [99] mentioned blinding, of which only 2 [48], [94] reported that they blinded outcome assessors, the other 2 trials did not report who were blinded. Five trials [11], [39], [72], [80], [116] reported the number of dropouts, but none of these used intention-to-treat analysis.

There were 101 (74.81%) trials that reported comparability of baseline data, 42 (31.11%) trials specified the inclusion criteria, 40 (29.63%) trials specified the exclusion criteria, and 101 (74.81%) trials described diagnostic criteria. Efficacy standard was reported in 126 (93.33%) trials, but 110 of them used composite outcome measures, which categorized treatment efficacy into four grades (cured, markedly effective, effective, and ineffective) according to change in symptoms, the other 16 trials used single outcome measure for therapeutic effect. Symptoms were commonly used as outcome measures.

### Estimate Effects of RCTs with Cupping

Due to insufficient number of RCTs and the variations in study quality, participants, intervention, variable control, and outcome measures, results of most of the studies could not be synthesized by quantitative methods. Though 133 of the 135 included studies showed that cupping therapy as well as cupping combined with other treatment were significantly effective for certain diseases (Table S6), interpretation of the positive findings from the individual studies needs to be incorporated with the clinical characteristics of the included studies and evidence power. Therefore, the beneficial effect of cupping therapy needs to be confirmed through large and rigorously-designed RCTs.

We conducted meta-analyses to evaluate therapeutic effect of cupping therapies for herpes zoster, facial paralysis, acne, and cervical spondylosis (Tables 2–5).

**Table 2:** Effect of estimates of wet cupping treatment for herpes zoster in 15 RCTs.

Trials	Comparisons	Effect estimates ([95%CI])	*P*
**Numbers of cured patients**			
*Wet cupping plus other interventions versus other interventions alone*			
*Wet cupping plus medication versus medications alone*			
Guo L 2006 [32]	Wet cupping plus aciclovir, VitB_1_, VitB_12_ versus aciclovir, VitB_1_, VitB_12_	RR 1.48 [1.05, 2.09]	
Liu L 2003 [61]	Wet cupping plus aciclovir, VitB_1_, VitB_12_ and aciclovir cream versus aciclovir, VitB_1_, VitB_12_, and aciclovir cream	RR 3.83 [2.07, 7.06]	
Long W 2003 [64]	Wet cupping plus ultraviolet radiation versus ultraviolet radiation alone	RR 1.30 [1.06, 1.59]	
Xu L 2004 [104]	Wet cupping plus aciclovir cream, aciclovir 0.5 g and glucose 250 ml intravenous drip versus aciclovir cream, aciclovir 0.5 g and glucose 250 ml intravenous drip	RR 1.35 [0.93, 1.97]	
Zhang Q 2008 [126]	Wet cupping and bloodletting on ear apex plus aciclovir and acupuncture versus aciclovir and acupuncture	RR 4.17 [1.92, 9.05]	
**Subgroup**		RR 1.93 [1.23, 3.04]*	**0.005**
*Wet cupping plus acupuncture versus acupuncture alone*			
Huang J 2008 [37]	Wet cupping plus acupuncture versus acupuncture alone	RR 2.38 [1.10, 5.13]	
Zhang H 2009 [122]	Wet cupping plus electroacupuncture versus electroacupuncture alone	RR 1.29 [0.95, 1.76]	
Zuo R 2010 [145]	Wet cupping plus electroacupuncture versus electroacupuncture alone	RR 1.91 [1.07, 3.42]	
**Subgroup**		**RR 1,65 [1.08, 2.53]**	**0.02**
**Overall (Random, ** ***I^2^*** ** = 76%)**		**RR 1.81 [1.33, 2.45]**	**0.0001**
*Wet cupping versus medications*			
Ci H 2010 [20]	Wet cupping versus aciclovir	RR 1.60 [1.24, 2.06]	
Jin M 2008 [45]	Wet cupping versus aciclovir, cimetidine, indomethacin, mecobalamin, calamine, and aciclovir cream	RR 2.15 [1.54, 3.00]	
Liu L 2003 [61]	Wet cupping versus aciclovir, VitB_1_, VitB_12_, and aciclovir cream	RR 2.83 [1.47, 5.46]	
Liu Q 2004 [63]	Wet cupping versus aciclovir and poly I-C injection	RR 2.90 [1.71, 4.91]	
Wang Y 2009 [94]	Wet cupping versus valaciclovir	RR 2.34 [1.66, 3.30]	
**Overall (Fixed, ** ***I^2^*** ** = 43%)**		**RR 2.07 [1.77, 2.43]**	**<0.00001**
**Numbers of patients with postherpetic neuralgia after treatment**			
*wet cupping versus medications alone*			
Jin M 2008 [45]	Wet cupping versus acyclovir, cimetidine, indomethacin, mecobalamin, 23acyclovir, andacyclovir cream	RR 0.09 [0.01, 1.60]	
Liu L 2003 [61]	Wet cupping versus acyclovir, VitB_1_, VitB_12_, and acyclovir cream	RR 0.06 [0.00, 1.09]	
Wang Y 2009 [94]	Wet cupping versus valaciclovir	RR 0.23 [0.08, 0.64]	
Xiong Z 2007 [103]	Wet cupping versus acyclovir plus normal saline 250 ml intravenous drip	RR 0.05 [0.01, 0.38]	
**Overall (Fixed, ** ***I^2^*** ** = 0%)**		**RR 0.12 [0.06, 0.28]**	**<0.00001**
**Numbers of patients with effective symptoms after treatment**			
*Wet cupping plus other interventions versus other interventions alone*			
*Wet cupping plus medication versus medication alone*			
Guo L 2006 [32]	Wet cupping plus aciclovir, VitB_1_, VitB_12_ versus aciclovir, VitB_1_, VitB_12_	RR 1.00 [0.92, 1.08]	
Liu L 2003 [61]	Wet cupping plus aciclovir, VitB_1_, VitB_12_ and aciclovir cream versus aciclovir, VitB_1_, VitB_12_, and aciclovir cream	RR 1.00 [0.95, 1.05]	
Xu L 2004 [104]	Wet cupping plus aciclovir cream, aciclovir 0.5 g and glucose 250 ml intravenous drip versus aciclovir cream, aciclovir 0.5 g, and glucose 250 ml intravenous drip	RR 1.00 [0.95, 1.05]	
Zhang Q 2008 [126]	Wet cupping and blood-letting on auditive apex plus aciclovir and acupuncture versus aciclovir and acupuncture	RR 1.00 [0.95, 1.05]	
**Subgroup**		**RR 1.00 [0.97, 1.03]**	**0.99**
*Wet cupping plus acupuncture versus acupuncture alone*			
Huang J 2008 [37]	Wet cupping plus acupuncture versus acupuncture alone	RR 1.09 [0.83, 1.43]	
Zhang H 2009 [122]	Wet cupping plus electroacupuncture versus electroacupuncture alone	RR 1.20 [0.97, 1.48]	
Zuo R 2010 [145]	Wet cupping plus electroacupuncture versus electroacupuncture alone	RR 1.11 [0.98, 1.27]	
**Subgroup**		**RR 1.13 [1.02, 1.25]**	**0.02**
**Overall (Random, ** ***I^2^*** ** = 52%)**		**RR 1.02 [0.98, 1.06]**	**0.41**
*Wet cupping versus medications*			
Ci H 2010 [20]	Wet cupping versus aciclovir	RR 1.22 [1.07, 1.40]	
Jin M 2008 [45]	Wet cupping versus aciclovir, cimetidine, indomethacin, mecobalamin, calamine, and aciclovir cream	RR 1.07 [0.98, 1.17]	
Liu L 2003 [61]	Wet cupping versus aciclovir, VitB_1_, VitB_12_ and aciclovir cream	RR 1.00 [0.94, 1.06]	
Liu Q 2004 [63]	Wet cupping versus aciclovir and poly I-C injection	RR 1.27 [1.05, 1.54]	
Wang Y 2009 [94]	Wet cupping versus valaciclovir	RR 1.08 [0.99, 1.17]	
**Overall (Random, ** ***I^2^*** ** = 82%)**		**RR 1.11 [1.00, 1.23]**	**0.06**
**Average cure time**			
*Wet cupping plus other interventions versus other interventions alone*			
Guo L 2006 [32]	Wet cupping plus aciclovir, VitB_1_, VitB_12_ versus aciclovir, VitB_1_, VitB_12_	MD −2.10 [−3.55, −0.65]	
Liu L 2003 [61]	Wet cupping plus aciclovir, VitB_1_, VitB_12_, and aciclovir cream versus aciclovir, VitB_1_, VitB_12_,and aciclovir cream	MD −5.08 [−8.04, −2.12]	
**Overall (Fixed, ** ***I^2^*** ** = 68%)**		**MD −2.67 [−3.97, −1.37]**	**<0.0001**
*Wet cupping versus medications*			
Liu L 2003 [61]	Wet cupping versus aciclovir, VitB_1_, VitB_12_, and aciclovir cream	MD −3.14 [−6.45, 0.17]	
**Overall (Fixed, ** ***I^2^*** ** = 0%)**		**MD −3.14 [−6.45, 0.17]**	**0.06**

**Table 3:** Effect of estimates of cupping for facial paralysis in 15 RCTs.

Trials	Comparisons	Effect Estimates ([95%CI])	*P*
**Numbers of cured patients**			
*Cupping plus other interventions versus other interventions alone*			
*Flash cupping plus acupuncture versus acupuncture alone*			
Cao R 2009 [12]	Flash cupping plus acupuncture versus acupuncture alone	RR 2.00 [1.09, 3.66]	
Fu C 2004 [25]	Flash cupping plus acupuncture versus acupuncture alone	RR 1.73 [1.30, 2.30]	
Huang L 2009 [39]	Flash cupping plus acupuncture versus acupuncture alone	RR 1.33 [0.95, 1.86]	
Li K 2009 [49]	Flash cupping plus acupuncture versus acupuncture alone	RR 1.50 [1.02, 2.21]	
Zhao N 2010 [133]	Flash cupping plus acupuncture versus acupuncture alone	RR 1.33 [1.04, 1.68]	
**Subgroup**		**RR 1.51 [1.29, 1.76]**	**<0.00001**
*Wet cupping plus acupuncture versus acupuncture alone*			
Gao B 2010 [28]	Wet cupping plus acupuncture and mecobalamine versus acupuncture and mecobalamine alone	RR 1.68[0.62, 4.53]	
Huang L 2010 [40]	Wet cupping plus acupuncture versus acupuncture alone	RR 1.60 [0.79, 3.23]	
Lü J 2010 [71]	Wet cupping plus acupuncture versus acupuncture alone	RR 1.29 [0.95, 1.76]	
Ren Y 2006 [77]	Wet cupping plus acupuncture versus acupuncture alone	RR 1.91 [1.32, 2.76]	
Sun H 2010 [80]	Wet cupping plus acupuncture versus acupuncture alone	RR 1.71 [1.23, 2.36]	
Wang L 2010 [89]	Wet cupping plus acupuncture versus acupuncture alone	RR 1.41 [0.85, 2.35]	
**Subgroup**		**RR 1.60 [1.33, 1.93]**	**<0.0001**
*Medicinal cupping plus medication versus medications*			
Qiu J 2003 [76]	Medicinal cupping plus neurotrophic drugs versus neurotrophic drugs alone	RR 1.44 [1.11, 1.87]	
**Subgroup**		**RR 1.44 [1.11, 1.87]**	**0.006**
*Wet cupping plus TDP and medications versus TDP and medications*			
Li W 2005 [51]	Wet cupping plus TDP, antivirus and neurotrophic drugs versus TDP and drugs alone	RR 1.18 [0.89, 1.57]	
**Subgroup**		**RR 1.18 [0.89, 1.57]**	**0.25**
*Flash cupping plus herbal medicine and acupuncture versus herbal medicine and acupuncture*			
Ou X 2009 [75]	Flash cupping plus herbal decoction and acupuncture versus herbal decoction and acupuncture	RR 1.37 [1.05, 1.80]	
**subgroup**		**RR 1.37 [1.05, 1.80]**	**0.02**
**Overall (Fixed, ** ***I^2^*** ** = 0%)**		**RR 1.49 [1.35, 1.65]**	**<0.00001**
*Wet cupping versus medications*			
Zhu F 2009 [141]	Wet cupping versus antivirus and neurotrophic drugs	RR 1.33 [0.83, 2.14]	
**Overall**		**RR 1.33 [0.83, 2.14]**	**0.23**

**Table 4:** Effect of estimates of cupping for acne in 6 RCTs.

Trials	Comparisons	Effect Estimates ([95%CI])	*P*
**Numbers of cured patients**			
*Wet cupping plus other interventions versus other interventions alone*			
*Wet cupping plus herbal medicine versus herbal medicine alone*			
Huang J 2010 [38]	Wet cupping plus herbal preparation, topical cream versus herbal preparation and external cream	RR 2.06 [1.33, 3.18]	
**Subgroup**		**RR 2.06 [1.33, 3.18]**	**0.001**
*Wet cupping plus acupuncture versus acupuncture alone*			
Liu H 2009 [59]	Flash cupping plus acupuncture versus acupuncture alone	RR 1.91 [0.99, 3.72]	
Wang Q 2007 [91]	Moving and wet cupping plus acupuncture versus acupuncture alone	RR 1.67 [0.87, 3.20]	
**Subgroup**		**RR 1,79 [1.12, 2.86]**	**0.01**
**Overall (Fixed, ** ***I^2^*** ** = 0%)**		**RR 1.93 [1.40, 2.65]**	**<0.0001**
*Wet cupping versus medications*			
Wu F 2010 [95]	Wet cupping versus tanshinone	RR 1.07 [0.45, 2.56]	
Wu Y 2008 [97]	Wet cupping versus tetracycline and ketoconazole cream	RR 2.50 [1.31, 4.77]	
Zhang K 2008 [125]	Wet cupping versus tetracycline	RR 2.75 [1.38, 5.48]	
**Overall (Fixed, ** ***I^2^*** ** = 37%)**		**RR 2.14 [1.42, 3.22]**	**0.0003**
**Average cure time**			
*Cupping plus other intervention versus other interventions alone*			
Li W 2005 [51]	Wet cupping plus TDP, antivirus and neurotrophic drugs versus TDP and drugs alone	MD −4.14 [−5.74, −2.54]	
Qiu J 2003 [76]	Medicinal cupping plus neurotrophic drugs versus neurotrophic drugs alone	MD −8.00 [−9.78, −6.22]	
**Overall (Random, ** ***I^2^*** ** = 90%)**		**MD −6.05 [−9.83, −2.27]**	**0.002**
*Wet cupping versus medications*			
Zhu F 2009 [141]	Wet cupping versus antivirus and neurotrophic drugs	MD −7.20 [−14.27, −0.13]	
**Overall**		**MD −7.20 [−14.27, −0.13]**	**0.05**

**Table 5:** Effect of estimates of cupping for cervical spondylosis in 6 RCTs.

Trials	Comparisons	Effect Estimates ([95%CI])	*P*
**Numbers of cured patients**			
*Cupping plus other interventions versus other interventions alone*			
*Cupping plus acupuncture versus acupuncture alone*			
Wan XW 2007 [86]	Needling cupping plus acupuncture versus acupuncture alone	RR 1.59 [1.14, 2.22]	
**Subgroup**		**RR 1.59 [1.14, 2.22]**	**0.007**
*Wet cupping plus acupuncture versus acupuncture alone*			
Shao M 2003 [79]	Wet cupping plus acupuncture versus acupuncture alone	RR 1.64 [1.04, 2.58]	
Wang PL 2010 [90]	Wet cupping plus acupuncture versus acupuncture alone	RR 1.15 [0.63, 2.12]	
**Subgroup**		**RR 1.46 [1.01, 2.09]**	**0.04**
*Wet cupping plus electroacupuncture versus electroacupuncture alone*			
Wang XM 2004 [93]	Wet cupping plus electroacupuncture versus electroacupuncture alone	RR 1.59 [0.72, 3.53]	
**Subgroup**		**RR 1.59 [0.72, 3.53]**	**0.25**
*Wet cupping plus traction versus traction alone*			
You Y 2006 [116]	Wet cupping plus traction versus traction alone	RR 1.55 [0.83, 2.91]	
**Subgroup**		**RR 1.55 [0.83, 2.91]**	**0.17**
**Overall (Fixed, ** ***I^2^*** ** = 0%)**		**RR 1.52 [1.20, 1.92]**	**0.0005**
**Numbers of effective patients**			
*Cupping plus other interventions versus other interventions alone*			
*Cupping plus acupuncture versus acupuncture alone*			
Wan XW 2007 [86]	Needling cupping plus acupuncture versus acupuncture alone	RR 10.36 [0.53, 201.45]	
**Subgroup**		**RR 10.36 [0.53, 201.45]**	**0.12**
*Wetcupping plus acupuncture versus acupuncture alone*			
Shao M 2003 [79]	Wet cupping plus acupuncture versus acupuncture alone	RR 2.90 [1.14, 7.38]	
Wang PL 2010 [90]	Wet cupping plus acupuncture versus acupuncture alone	RR 6.83 [0.79, 59.48]	
**Subgroup**		**RR 3.43 [1.47, 8.01]**	**0.004**
*Wet cupping plus electroacupuncture versus electroacupuncture alone*			
Wang XM 2004 [93]	Wet cupping plus electroacupuncture versus electroacupuncture alone	RR 7.22 [0.72, 72.56]	
**Subgroup**		**RR 7.22 [0.72, 72.56]**	**0.09**
*Wet cupping plus warm acupuncture versus warm acupuncture alone*			
Zeng HW 2007 [117]	Wet cupping plus acupuncture and moxibustion versus acupuncture and moxibustion alone	RR 3.86 [1.12, 13.26]	
**Subgroup**		**RR 3.86 [1.12, 13.26]**	**0.03**
*Wet cupping plus traction versus traction alone*			
You Y 2006 [116]	Wet cupping plus traction versus traction alone	RR 3.24 [1.04, 10.05]	
**Subgroup**		**RR 3.24 [1.04, 10.05]**	**0.17**
**Overall (Fixed, ** ***I^2^*** ** = 0%)**		**RR 3.84 [2.19, 6.75]**	**<0.00001**
*Wet cupping versus medications*			
Zeng HW 2007 [117]	Wet cupping versus flunarizine	RR 1.18 [0.60, 2.32]	
**Overall**		**RR 1.18 [0.60, 2.32]**	**0.63**

**Figure 2:**
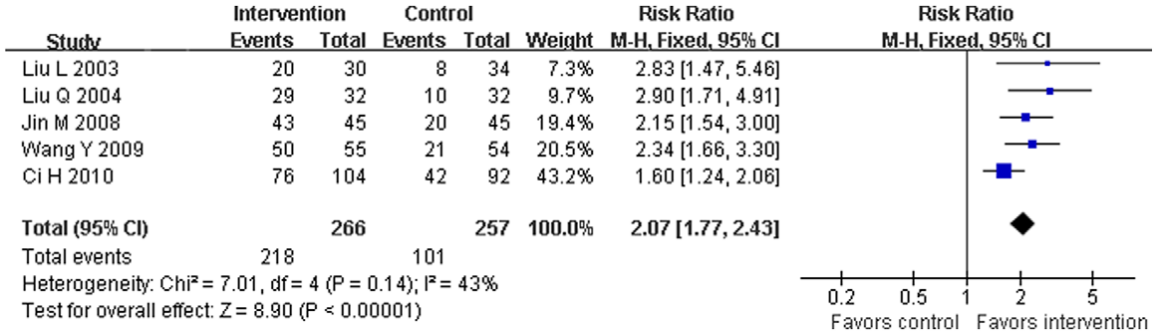
Effect of estimates of wet cupping versus medication on numbers of cured patients with herpes zoster.

**Figure 3:**
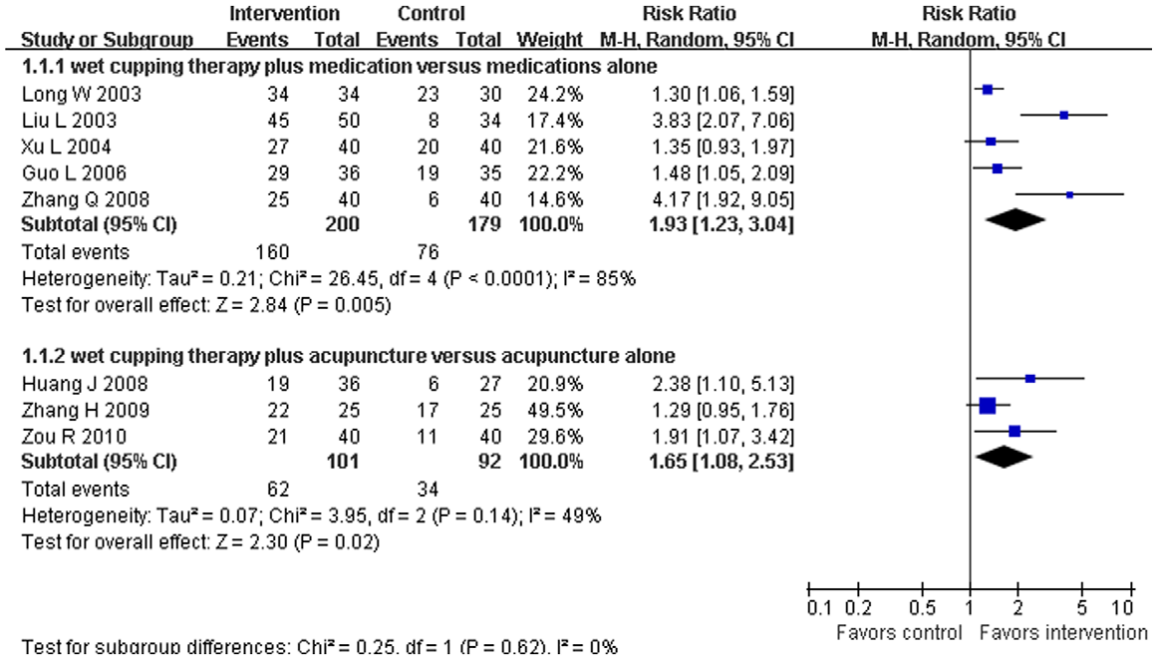
Effect of estimates of combination of wet cupping and other interventions versus other interventions alone on numbers of cured patients of herpes zoster.

Meta-analysis of 15 RCTs [20], [29], [32], [37], [45], [61], [63], [64], [94], [102]–[104], [122], [126], [145] to evaluate the efficacy of wet cupping therapy for herpes zoster (2 trials [13], [123] were excluded due to insufficient data), wet cupping was found to be superior to pharmaceutical medications, such as antiviral, in effecting a cure (RR 2.07, 95%CI 1.77 to 2.43, *p*<0.00001, 5 trials, random model) (Figure 2), and in lowering the incidence rate of post-herpetic neuralgia (RR 0.12, 95%CI 0.06 to 0.28, *p*<0.00001, 4 trials, fixed model). But no difference was identified in the number of patients with improved symptoms (RR 1.11, 95%CI 1.00 to 1.23, *p* = 0.06, 5 trials, random model). Wet cupping in combination with pharmaceutical medications was significantly better than medications alone in effecting a cure (RR 1.93, 95%CI 1.23 to 3.04, *p* = 0.005, 5 trials, random model), but no difference in symptom improvement was observed (RR 1.00, 95%CI 0.97 to 1.03, *p* = 0.99, 4 trials, random model) (Figure 3). Wet cupping combined with acupuncture was superior to acupuncture alone both in effecting a cure (RR 1.65, 95%CI 1.08 to 2.53, *p* = 0.02, 3 trials, random model) (Figure 3) and in improving symptoms (RR 1.13, 95%CI 1.02 to 1.25, *p* = 0.02, 3 trials, random model).

**Figure 4:**
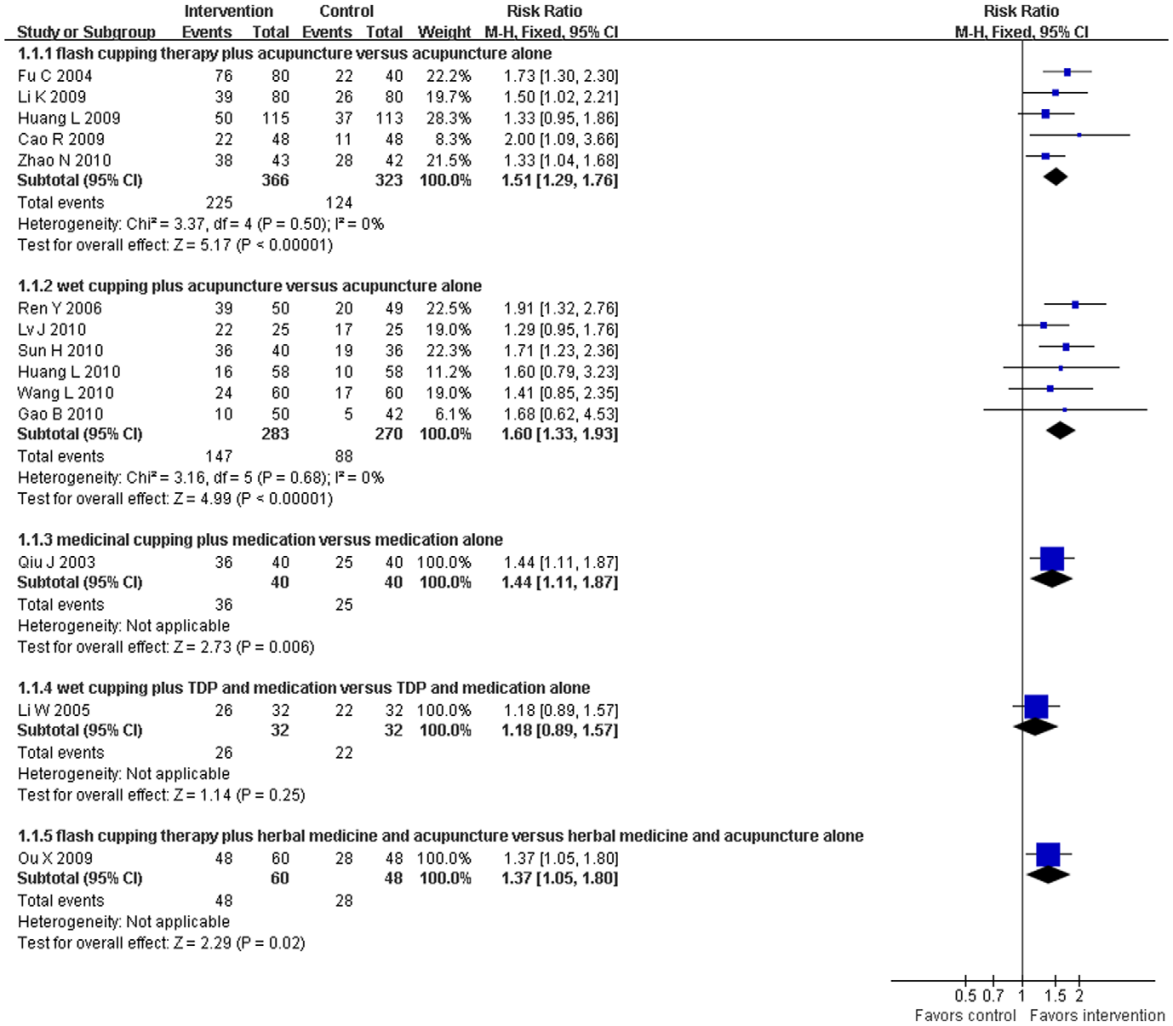
Effect of estimates of cupping combined with other interventions versus other interventions alone on numbers of cured patients with facial paralysis.

There were 17 RCTs [12], [15], [39], [40], [47], [49]–[51], [71], [75]–[77], [80], [89], [133], [141] that assessed the therapeutic effect of cupping therapy for facial paralysis. Two of the trials [47], [50] were excluded from the meta-analysis due to the incomparability between treatment and control groups. Six trials used flash cupping therapy, 8 trials used wet cupping, and 1 trial used medicinal cupping as the main intervention. Meta-analysis showed flash cupping combined with acupuncture (RR 1.51, 95%CI 1.29 to 1.76, *p*<0.00001, 5 trials, fixed model) and wet cupping combined with acupuncture (RR 1.60, 95%CI 1.33 to 1.93, *p*<0.0001, 6 trials, fixed model) were markedly better than acupuncture alone in effecting a cure (Figure 4). In addition, cupping in combination with medications, such as neurotrophic drugs, was superior to medications alone in reducing average cure time (MD −6.05, 95%CI −9.83 to −2.27, *p* = 0.002, 2 trials, random model).

Six trials [38], [59], [91], [95], [97], [125] evaluated the efficacy of cupping therapy for acne. Meta-analysis showed that, for improving the cure rate, wet cupping therapy was significantly better than medications, such as tanshinone, tetracycline, and ketokonazole (RR 2.14, 95%CI 1.42 to 3.22, *p* = 0.0003, 3 trials, fixed model). Furthermore, cupping therapy combined with other interventions was superior to other interventions alone (RR 1.93, 95%CI 1.40 to 2.65, *p*<0.0001, 3 trials, fixed model). As each comparison had less than five trials, it was not meaningful to conduct a funnel plot analysis.

For cervical spondylosis, 6 trials [79], [86], [90], [93], [116], [117] evaluated the efficacy of cupping therapy on this condition. Cupping therapy, especially wet cupping on GV-14 and *Ashi* points, combined with other treatment, including acupuncture and traction, was better than other treatments alone in effecting a cure (RR 1.52, 95%CI 1.20 to 1.92, *p* = 0.0005, 5 trials, fixed model) and in ameliorating symptoms (RR 3.84, 95%CI 2.19 to 6.75, *p*<0.00001, 6 trials, fixed model). One trial [117] compared wet cupping with flunarizine for symptom improvement, and found no difference between the two groups (RR 1.18, 95%CI 0.60 to 2.32, *p* = 0.63, 1 trial).

**Figure 5:**
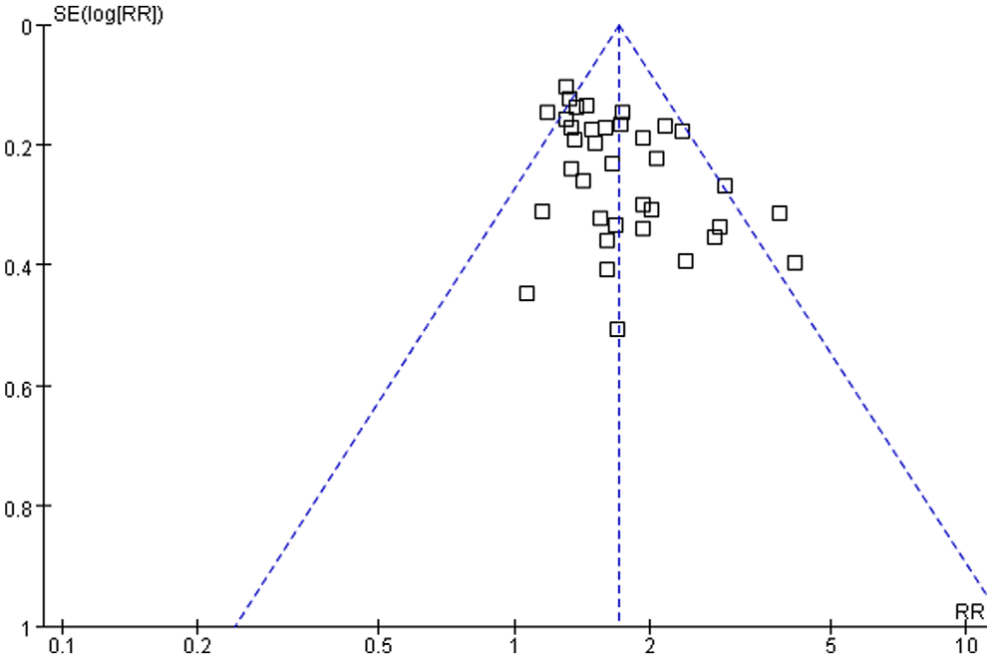
Funnel plot assessing outcomes of cured patients reported in 39 randomized controlled trials on 4 diseases.

A funnel plot analysis of 39 trials was performed to examine outcome for the number of cured patients irrespective of disease. The result showed potential asymmetry (Figure 5).

Serious adverse effects were not reported in any of the 135 included trials.

## Discussion

In our previous review [3], we focused on the characteristics of the RCTs on cupping therapy. This review aimed to ascertain whether or not cupping therapy is efficacious for several conditions, especially when combined with other treatments. With this review, we expanded our search to include articles published from 2008 through 2010. The 62 new studies indicate that the ancient TCM practice of cupping remains an important therapeutic modality in China and is gaining recognition elsewhere. For diseases/conditions that are commonly treated by cupping, we conducted meta-analyses by synthesizing data from homogeneous studies to assess the therapeutic effect of cupping in treating these diseases/conditions. For studies whose data were inappropriate for synthesis, we used qualitative methods to evaluate their findings. This is the first instance that quantitative and qualitative methods were used in a systematic review to evaluate the efficacy of cupping therapy.

Despite the large number of studies on cupping therapy, including the 62 new ones, there remains a lack of well-designed investigations. Of the 135 RCTs included in this review, 84.44% were high risk of bias. One issue is adherence to the Consolidated Standards of Reporting Trials (CONSORT) [146] in which randomization methods should be clearly described and fully reported. Another issue is blinding, which continues to be a challenge for studies involving manual healing therapies, such as acupuncture, massage, and cupping therapy. Lee et al [147] report developing a sham cupping device with a tiny opening that in effect reduces the negative pressure in the cup once it is attached to the skin. The RCT they conducted showed that the device appears to be tenable as a control for actual cupping, though confirmatory studies are needed. While blinding during studies on cupping therapy may be difficult to achieve, at the very least, blinding of outcome assessors and statistics should be attempted to minimize performance and assessment biases. Another area that researchers should be attentive to is adapting STRICTA [148] standards when designing and reporting studies. Similar to acupuncture, cupping therapy is based on energy channels (meridians) and acupoints. Therefore, methodology details should be reported, including types of cups, acupoints used and their TCM rationale, practitioner background, number of treatment sessions and frequency, among other STRICTA-recommended information. Standardization can also be achieved by registering with and following the protocol of international organizations [149], such as WHO International Clinical Trials Registry Platform (ICTRP) [150].

As in our previous review, we continue to emphasize the importance of using standard outcome measures for specific diseases/conditions. As mentioned, 80.74% of the included trials used composite outcome measures, which categorized treatment efficacy into four grades. The classifications of “cure,” “markedly effective,” “effective,” and “ineffective” are not internationally recognized with their exact meaning open to interpretation. This can increase clinical heterogeneity. We suggest that researchers comply with international standards, such as the House Brackmann score for facial nerve paralysis (Bell palsy), in the evaluation of treatment efficacy to give credibility to their work.

The potential asymmetry of the overall funnel plot test (Figure 5) of 39 RCTs that examined the outcome of the number of cured patients for 4 diseases (herpes zoster, facial paralysis, acne, and cervical spondylosis) may be caused by, small study effects, or even heterogeneity in intervention effects. Furthermore, as we did not include unpublished studies, there is high potential that our review may have publication bias. We strongly recommend that researchers plan their sample size for randomized controlled trials to ensure adequate statistical power. Furthermore, sample size calculation and analysis of outcomes should be based on the principle of intention-to-treat.

Finally, our meta-analysis revealed that cupping therapy combined with other treatments, such as acupuncture or medications, showed significant benefit over other treatments alone in effecting a cure for herpes zoster, acne, facial paralysis, and cervical spondylosis. This appears to support the common practice in China of combining TCM therapeutic modalities, either TCM with TCM, or TCM with routine western medicine, to enhance efficacy. The effect of cupping therapy over time is not known, but use of cupping is generally safe based on long-term clinical application and outcomes reported in the reviewed trials.

In conclusion, the results of this systematic review suggest that cupping therapy appears to be effective for various diseases/conditions, in particular herpes zoster, acne, facial paralysis, and cervical spondylosis. However, the main limitation of our analysis was that nearly all included trials were evaluated as high risk of bias. As such, it is necessary to conduct further RCTs that are of high quality and larger sample sizes in order to draw a definitive conclusion.

## Supporting Information

Table S1 **Mapping of diseases/conditions reported in cupping trials (1992–2010).**
(DOC)

Table S2 **Characteristics of 15 included trials on cupping for herpes zoster.**
(DOC)

Table S3 **Characteristics of 15 included trials on cupping for facial paralysis (Bell palsy).**
(DOC)

Table S4 **Characteristics of 6 included trials on cupping for acne.**
(DOC)

Table S5 **Characteristics of 6 included trials on cupping for cervical spondylosis.**
(DOC)

Table S6 **Characteristics of randomized controlled trials outside meta-analysis.**
(DOC)

Checklist S1 **CONSORT checklist.**
(DOC)

Protocol S1 **Flow chart of search strategy for inclusion and exclusion of studies.**
(DOC)
