# Supplementary material for: An Updated Review of the Efficacy of Cupping Therapy
Source: PLoS One. 2012 Feb 28;7(2):e31793. doi: 10.1371/journal.pone.0031793 (PMC3289625; doi:10.1371/journal.pone.0031793)
Supplement: Table S1 — Mapping of diseases/conditions reported in cupping trials (1992–2010). (DOC) [file pone.0031793.s001.doc]

**Table S1 Mapping of diseases/conditions reported in cupping trials (1992 – 2010)**

| **Disease** | **Total participants** | **Year** | | | | | | | | | | | | | | | | | | | **Total**  **trials** |
| --- | --- | --- | --- | --- | --- | --- | --- | --- | --- | --- | --- | --- | --- | --- | --- | --- | --- | --- | --- | --- | --- |
|  |  | **92** | **93** | **94** | **95** | **96** | **97** | **98** | **99** | **00** | **01** | **02** | **03** | **04** | **05** | **06** | **07** | **08** | **09** | **10** |  |
| Herpes zoster (post- herpetic neuralgia) | **1439** |  | 1 |  |  |  |  |  |  |  |  |  | 2 | 3 |  | 1 | 2 | 3 | 3 | 2 | **17** |
| Facial paralysis (Bell palsy) | **1814** |  |  |  |  |  |  |  |  |  |  |  | 1 | 1 | 2 | 1 |  |  | 5 | 7 | **17** |
| Symptom of cough/dyspnea | **1433** |  |  |  |  |  |  |  | 1 |  |  |  |  | 1 | 1 | 2 |  |  | 1 | 2 | **8** |
| Acne | **472** |  |  |  |  |  |  |  |  |  |  |  |  |  |  |  | 1 | 2 | 1 | 2 | **6** |
| Prolapse of lumbar intervertebral disc | **471** |  |  |  |  |  |  |  |  |  |  |  |  |  | 1 | 1 | 1 | 1 | 1 | 1 | **6** |
| Cervical spondylosis | **602** |  |  |  |  |  |  |  |  |  |  |  | 1 | 1 |  | 1 | 2 |  |  | 1 | **6** |
| Lumbar muscle strain | **584** |  |  |  |  |  |  |  |  |  |  |  |  |  |  |  |  |  | 2 | 3 | **5** |
| Obesity | **503** |  |  |  |  |  |  |  |  |  |  |  |  |  |  | 2 | 1 | 1 |  |  | **4** |
| Wound and abscess | **711** |  |  |  |  |  | 1 | 1 | 1 |  |  |  |  |  | 1 |  |  |  |  |  | **4** |
| Nonspecific low back pain | **243** |  |  |  |  |  |  |  |  |  |  |  |  |  |  | 1 |  | 1 | 1 |  | **3** |
| Chloasma | **258** |  |  |  |  |  |  |  |  |  |  |  |  |  |  | 1 |  |  | 1 | 1 | **3** |
| Hand-shoulder syndrome | **190** |  |  |  |  |  |  |  |  |  |  |  |  |  |  |  |  |  | 3 |  | **3** |
| Chronic obstructive pulmonary disease | **222** |  |  |  |  |  |  |  |  |  |  |  |  |  |  |  |  |  | 1 | 2 | **3** |
| Common cold | **214** |  |  | 1 |  |  |  |  |  |  |  |  |  | 1 |  |  |  |  |  |  | **2** |
| Scapulohumeral periarthritis | **178** |  |  |  |  |  |  |  |  |  |  |  |  |  |  |  |  |  | 1 | 1 | **2** |
| Insomnia | **140** |  |  |  |  |  |  |  |  |  |  |  |  |  |  |  | 1 |  | 1 |  | **2** |
| Nausea/vomiting | **92** |  |  |  |  |  |  |  |  |  |  |  |  |  |  |  |  |  | 2 |  | **2** |
| Erysipelas | **157** |  |  |  |  |  |  |  |  |  |  |  |  |  | 1 | 1 |  |  |  |  | **2** |
| Osteoarthritis | **215** |  |  |  |  |  |  |  |  |  |  |  |  |  |  |  |  | 2 |  |  | **2** |
| Vertigo | **189** |  |  |  |  |  |  |  |  |  |  |  |  |  |  |  |  |  | 2 |  | **2** |
| Leucoderma | **120** | 1 |  |  |  |  |  |  |  |  |  |  |  |  |  |  |  |  |  |  | **1** |
| Brachialgia paraesthetica nocturna | **20** |  |  |  |  |  |  |  |  |  |  |  |  |  |  | 1 |  |  |  |  | **1** |
| Venomous snake bite | **100** |  |  |  |  |  |  |  |  |  |  |  |  |  |  |  |  |  |  | 1 | **1** |
| Atherosclerosis | **42** |  |  |  |  |  |  |  |  |  |  |  |  |  |  |  | 1 |  |  |  | **1** |
| Cancer pain | **30** |  |  |  |  |  |  |  |  |  |  |  |  |  |  | 1 |  |  |  |  | **1** |
| External humeral epicondylitis | **100** |  |  |  |  |  |  |  |  |  |  |  |  |  |  |  |  |  |  | 1 | **1** |
| Functional dyspepsia | **75** |  |  |  |  |  |  |  |  |  |  |  |  |  |  |  |  |  |  | 1 | **1** |
| Lateral femoral cutaneous neuritis | **148** |  |  |  |  |  |  |  |  |  |  |  |  |  |  | 1 |  |  |  |  | **1** |
| Acute ankle sprain | **92** |  |  |  |  |  |  |  |  |  |  |  |  |  |  |  | 1 |  |  |  | **1** |
| Pain in low back and lower extremities | **87** |  |  |  |  |  |  |  |  | 1 |  |  |  |  |  |  |  |  |  |  | **1** |
| Ulcerative colitis | **45** |  |  |  |  |  |  |  |  | 1 |  |  |  |  |  |  |  |  |  |  | **1** |
| Schizophrenia | **60** |  |  |  |  |  | 1 |  |  |  |  |  |  |  |  |  |  |  |  |  | **1** |
| Intracranial hypertension | **80** |  |  |  |  |  |  |  |  |  |  |  | 1 |  |  |  |  |  |  |  | **1** |
| Rotaviral enteritis | **150** |  |  |  |  |  |  |  |  |  |  |  |  |  | 1 |  |  |  |  |  | **1** |
| Chronic diarrhea | **60** |  |  |  |  |  |  |  |  |  |  |  |  |  |  | 1 |  |  |  |  | **1** |
| Chronic urticaria | **60** |  |  |  |  |  |  |  |  |  |  |  |  |  |  |  |  |  |  | 1 | **1** |
| Facial spasm | **48** |  |  |  |  |  |  |  |  |  |  |  |  |  |  |  |  |  |  | 1 | **1** |
| Contusion of temporomandibular joint | **41** |  |  |  |  |  |  |  |  |  |  |  |  |  |  | 1 |  |  |  |  | **1** |
| Spondylitis ankylopoietica | **62** |  |  |  |  |  |  |  |  |  |  |  |  |  | 1 |  |  |  |  |  | **1** |
| Edema of the upper extremity after surgery | **67** |  |  |  |  |  |  |  |  |  |  |  |  |  |  |  |  | 1 |  |  | **1** |
| Acute mastitis | **200** | 1 |  |  |  |  |  |  |  |  |  |  |  |  |  |  |  |  |  |  | **1** |
| Soft tissue injury | **307** |  |  |  |  |  |  |  |  |  |  |  |  | 1 |  |  |  |  |  |  | **1** |
| Depression | **115** |  |  |  |  |  |  |  |  |  |  |  |  |  |  | 1 |  |  |  |  | **1** |
| Post-operative retention of urine | **120** |  |  |  |  |  |  |  |  |  |  |  |  |  |  | 1 |  |  |  |  | **1** |
| Diabetic peripheral neuropathy | **65** |  |  |  |  |  |  |  |  |  |  |  | 1 |  |  |  |  |  |  |  | **1** |
| Carpal tunnel syndrome | **52** |  |  |  |  |  |  |  |  |  |  |  |  |  |  |  |  |  | 1 |  | **1** |
| Perimenopausal syndrome | **60** |  |  |  |  |  |  |  |  |  |  |  |  |  | 1 |  |  |  |  |  | **1** |
| Knee pain | **109** |  |  |  |  |  |  |  |  |  |  |  |  |  | 1 |  |  |  |  |  | **1** |
| Heatstroke | **60** |  |  |  |  |  |  |  |  |  |  |  |  |  |  | 1 |  |  |  |  | **1** |
| Nonspecific subclinical disease | **62** |  |  |  |  |  |  |  |  |  |  |  |  |  |  |  |  | 1 |  |  | **1** |
| Psoriasis | **72** |  |  |  |  |  |  |  |  |  |  |  |  |  |  |  |  |  | 1 |  | **1** |
| Sciatica | **86** |  |  |  |  |  |  |  |  |  |  |  | 1 |  |  |  |  |  |  |  | **1** |
| Myofasciitis of the upper back | **90** |  |  |  |  |  |  |  |  |  |  |  |  |  |  |  | 1 |  |  |  | **1** |
| Inflammation of superior cluneal nerves | **155** |  |  |  |  |  |  |  |  |  |  |  |  |  |  |  |  |  |  | 1 | **1** |
| Stroke | **80** |  |  |  |  |  |  |  |  |  |  |  |  |  |  |  |  |  | 1 |  | **1** |
| Chronic gastritis | **112** |  |  |  |  |  |  |  |  |  |  |  |  |  |  |  |  |  |  | 1 | **1** |
| **Total number** | **13359** | **2** | **1** | **1** | **0** | **0** | **2** | **1** | **2** | **2** | **0** | **0** | **7** | **8** | **10** | **19** | **11** | **12** | **28** | **29** | **135** |
