# Supplementary material for: An Updated Review of the Efficacy of Cupping Therapy
Source: PLoS One. 2012 Feb 28;7(2):e31793. doi: 10.1371/journal.pone.0031793 (PMC3289625; doi:10.1371/journal.pone.0031793)
Supplement: Table S2 — Characteristics of 15 included trials on cupping for herpes zoster. (DOC) [file pone.0031793.s002.doc]

**Table S2 Characteristics of 15 included trials on cupping for herpes zoster**

| **Trials** | **Patients (M/F)** | | **Average age (y)** | **Diagnostic**  **criteria** | **Interventions** | | | **Duration of treatment** | **Outcome measure** |
| --- | --- | --- | --- | --- | --- | --- | --- | --- | --- |
|  | **Treatment** | **Control** |  |  | **Cupping treatment** | **Control** | |  |  |
| Ci H 2010 [20] | 48/56 | 42/50 | 51.6 | Medical textbook published in China: *Modern Dermatology* | Prick lesion with standard acupuncture needle, followed by cupping on the same site for 10 minutes, once every 2 days | Aciclovir 0.5 g plus 250 ml normal saline intravenous drip twice daily | | 12 days | **Cured, markedly effective, ineffective |
| Gao Y 2009 [29] | 19/11 | 17/10 | 45.6 | Not reported | Prick lesion with tri-ensiform needle followed by cupping on same site for 10 minutes, once every two days, plus electroacupuncture for 30 minutes, once daily | Carbamazepine 0.1 g three times daily, mecobalamine 500 μg twice daily, plus electroacupuncture for 30 minutes once daily | | 20 days | Pain relieved |
| Guo L 2006 [32] | 19/17 | 17/18 | Not reported | Medical textbook published in China: *Routine Diagnostics* | Prick lesion with tri-ensiform needle followed by cupping on same site for 10 minutes, once every 2 days, plus aciclovir 200 mg 3 times daily, vitamin B1 100 mg, vitamin B12 250 mg injection once daily | Aciclovir 200 mg 3 times daily, vitamin B1 100 mg, vitamin B12 250 mg injection once daily | | 10 days | *Cured, improved, ineffective; average time of cure |
| Huang J 2008 [37] | 11/25 | 9/18 | 58.6 | Medical textbook published in China: *Dermatology* | Tap lesion with plum blossom needle followed by cupping on same site for 2-3 minutes, once daily, plus routine body acupuncture 30 minutes once daily | Routine body acupuncture for 30 minutes once daily, using the same acupoints as for the treatment group | | 10 days | *Cured, improved, ineffective |
| Jin M 2008 [45] | 26/19 | 25/20 | 55.5 | TCM practice guideline for diagnosis and defining treatment efficacy | Tap lesion with plum blossom needle followed by cupping on the same site for 10-15 minutes, once daily for 3 days, then once every 2 days, and finally for 4 days | Aciclovir 0.2 g five times daily, cimetidine 0.2 g three times daily, indomethacin 50 mg three times daily, mecobalamin 0.5 mg three times daily, apply calamine lotion and aciclovir cream (strength not reported) | | 10 days | *Cured, improved, ineffective;  incidence rate of postherpetic neuralgia |
| Lin L 2003 [61] | Group 1  28/22 | Group 1  19/15 | Group 1  55.1 | Medical textbook published in China---*Dermatology* | Group1: Tap lesion with plum blossom needle followed by cupping on the same site for 10-15 minutes, once every 2 days, plus aciclovir 0.2 g five times daily, vitamin B1 20 mg three times daily, vitamin B12 500 mg injection once every 2 days, 2%-3% aciclovir cream for topical use | Group 1: Aciclovir 0.2 g five times daily, vitamin B1 20 mg three times daily, vitamin B12 500 mg injection once every 2 days, 2%-3% aciclovir cream for topical use | | 10 days | **Cured, markedly effective, effective, ineffective;  average time of cure;  incidence rate of postherpetic neuralgia |
|  | Group 2  16/14 | Group 2  19/15 | Group 2  54.2 |  | Group 2: Tap lesion with plum blossom needle followed by cupping on the same site for 10-15 minutes, once every 2 days | Group 2: Aciclovir 0.2 g five times daily, vitamin B1 20 mg three times daily, vitamin B12 500 mg injection once every 2 days, 2%-3% aciclovir cream for topical use | |  |  |
| Liu Q 2004 [63] | 32 (gender proportion not reported) | 32 (gender proportion not reported) | 55.6 | Not reported | Prick lesion with tri-ensiform needle followed by cupping on lesion, frequency not reported | Aciclovir 1.2 g five times daily, poly I:C injection 2 mg once every 2 days | 10 days | | **Cured, markedly effective, effective, ineffective |
| Long W 2003[64] | 34 (gender proportion not reported) | 30 (gender proportion not reported) | 44.5 | Not reported | Tap lesion with plum blossom needle followed by cupping on the same site, plus ultraviolet radiation once every 2 days | Ultraviolet radiation once every 2 days | 10 days | | Times of treatment for *cured, |
| Wang Y 2009 [94] | 55 (gender proportion not reported) | 54 (gender proportion not reported) | Not reported | Medical textbook published in China: *Modern Dermatology* | Routine acupuncture needling on adjacent to lesion followed by cupping on the same site for 5-10 minutes, once daily for 3 days, then once every 2 days | Valaciclovir 0.3 g twice daily | 9 days | | * Improved, ineffective;  self-report by patients of their symptoms;;  average time for lesions to crust over; average time for pain to resolve |
| Xiong S 2004 [100] | 56 (gender proportion not reported) | 56 (gender proportion not reported) | 52 | TCM practice guideline for diagnosis and defining treatment efficacy | Tap lesion with plum blossom needle followed by cupping on the same site for 10-15 minutes, once daily, plus herbal decoction twice daily | Herbal decoction twice daily | Until pain resolves | | Average time for pain to resolve |
| Xiong Z 2007 [103] | 20/28 | 16/24 | 49 | Medical textbook published in China: *Clinical Dermatology* | Prick lesion followed by cupping for 5 minutes, frequency not reported | Aciclovir (dosage not reported) plus 250 ml normal saline intravenous drip once daily, | 7 days | | Incidence rate of postherpetic neuralgia |
| Xu L 2004 [104] | 20/20 | 21/19 | Not reported | Not reported | Prick lesion with tri-ensiform needle followed by cupping on same site for 15 minutes once daily, plusacyclovir cream for topical use (strength not reported) plus acyclovir 0.5 g oral, and glucose 250 ml intravenous drip twice daily | Aciclovir cream (strength not reported) for topical use, plus acyclovir 0.5 g and glucose 250 ml intravenous drip twice daily | 7 days | | *Cured, markedly effective, improved, ineffective;  self-report of patients of their symptoms;  average time for lesions to crust over; average time for pain to resolve |
| Zhang H 2009 [122] | 10/15 | 12/13 | Not reported | TCM practice guideline for diagnosis and defining treatment efficacy | Tap lesion with plum blossom needle followed by cupping on same site for 5-10 minutes, plus electroacupuncture for 30 minutes, once daily | Electroacupuncture for 30 minutes once daily | 10 days | | *Cured, markedly effective, improved, ineffective;  pain resolved;  average duration of pain |
| Zhang Q 2008 [126] | 14/26 | 12/28 | Not reported | Medical textbook published in China: *Clinical Dermatology* | Routine acupuncture needling adjacent tothe lesion 30 minutes once daily, plus prick with tri-ensiform needle GV14, bilateral BL13 and BL18 , apply cupping for 10 minutes once every 2 days andblood-letting ear apex twice aweek, aciclovir 200 mg five times daily | Aciclovir 200 mg five times daily, routine acupuncture needling adjacent to the lesion 30 minutes once daily | 14 days | | *Cured, markedly effective, improved, ineffective |
| Zou R 2010 [145] | 14/26 | 13/27 | 43.9 | TCM practice guideline for diagnosis and defining treatment efficacy | Tap lesion with plum blossom needle followed by cupping on the same site for 5-10 minutes, plus electroacupuncture for 30 minutes once daily | Electroacupuncture for 30 minutes once daily | 10 days | | *Cured, markedly effective, improved, ineffective;  average time for pain to resolve; average time for lesion to crust over |

Definitions of “cured,” “markedly effective,” “effective,” and “ineffective”:

*Cured: Rash completely subsided; clinical symptoms, including pain, are resolved.

Markedly effective: Rash more than 70% subsided; clinical symptoms, including pain, are nearly resolved.

Improved: Rash 30%-69% subsided; clinical symptoms, including pain, are effective.

Ineffective: Rash less than 30% subsided; clinical symptoms, including pain, are not relieved.

**Cured: Rash completely subsided; clinical symptoms, including pain, are resolved.

Markedly effective: Rash more than 50% subsided; clinical symptoms, including pain, are nearly resolved.

Effective: Rash 10%-50% subsided; clinical symptoms, including pain, have been somewhat alleviated.

Ineffective: Rash less than 10% subsided; clinical symptoms, including pain, are not relieved.
