# Supplementary material for: An Updated Review of the Efficacy of Cupping Therapy
Source: PLoS One. 2012 Feb 28;7(2):e31793. doi: 10.1371/journal.pone.0031793 (PMC3289625; doi:10.1371/journal.pone.0031793)
Supplement: Table S3 — Characteristics of 15 included trials on cupping for facial paralysis (Bell palsy). (DOC) [file pone.0031793.s003.doc]

**Table S3 Characteristics of 15 included trials on cupping for facial paralysis (Bell palsy)**

| **Trials** | **Patients (M/F)** | | **Average age (y)** | **Diagnostic**  **criteria** | **Interventions** | | **Duration of treatment** | **Outcome measure** |
| --- | --- | --- | --- | --- | --- | --- | --- | --- |
|  | **Treatment** | **Control** |  |  | **Cupping treatment** | **Control** |  |  |
| Cao R 2009 [12] | 48 (gender proportion not reported) | 48 (gender proportion not reported) | Not reported | Medical textbook published in China:— *Neurology* | Flash cupping on relevant acupoints, plus routine body acupuncture for 30 minutes, once daily | Routine body acupuncture for 30 minutes once daily | 30 days | Cured, markedly effective, effective, ineffective |
| Fu C 2004 [15] | 80 (gender proportion not reported) | 40 (gender proportion not reported) | Not reported | Not reported | Flash cupping on relevant acupoints, cups retained in place for 10 minutes, plus routine body acupuncture for 30 minutes, once daily | Routine body acupuncture for 30 minutes once daily | 30 days | Cured, markedly effective, effective, ineffective based on symptom improvement |
| Gao B 2010 [28] | 26/24 | 22/20 | 39.4 | Medical textbook in China: *Neurology* | Prick relevant acupoints with tri-ensiform needle, followed by cupping on same acupoints for 10 minutes, once every 2 days, plus routine body acupuncture for 30 minutes once daily, and mecobalamine intramuscular injection (dosage not reported) once every 2 days | Routine body acupuncture for 30 minutes once daily, plus mecobalamine intramuscular injection (dosage not reported) once every 2 days | 20 days | Cured, markedly effective, effective, ineffective based on symptom improvement |
| Huang L 2009 [39] | 71/49 | 73/47 | 40.7 | Medical textbook published in China: *Neurology* | Flash cupping on relevant acupoints for 5 minutes plus routine body acupuncture for 30 minutes, once daily | Routine body acupuncture for 30 minutes once daily | 30 days | Cured, markedly effective, effective, ineffective based on symptom improvement |
| Huang L 2010 [40] | 27/31 | 30/28 | Not reported | TCM practice guideline for diagnosis and defining treatment efficacy | Prick relevant acupoints with standard needle, followed by cupping on same acupoints until 3 ml to 5 ml of blood are let during each cupping sessions for 5 consecutive days, plus routine body acupuncture for 30 minutes once daily | Routine body acupuncture for 30 minutes once daily | 30 days | Cured, markedly effective, effective, ineffective based on symptom improvement |
| Li K 2009 [49] | 80 (gender proportion not reported) | 80 (gender proportion not reported) | 40.2 | Medical textbook published in China: *Neurology* | Flash cupping on relevant acupoints for 5 minutes, plus routine body acupuncture for 30 minutes, once daily | Routine body acupuncture for 30 minutes once daily | 20 days | Cured, markedly effective, effective, ineffective based on symptom improvement |
| Li W 2005 [51] | 24/8 | 22/10 | 42.3 | — Medical textbook published in China: *Neurology* | Tap affected area with plum blossom needle, followed by cupping on relevant acupoints for 5 minutes once daily, plus TDP mineral lamp radiation for 20 minutes, and antiviral and neurotrophic medications once daily (types and dosage not reported) | TDP mineral lamp radiation for 20 minutes on the affected area, plus antiviral and neurotrophic medications once daily (types and dosage not reported) | 20 days | Cured, markedly effective, effective, ineffective based on symptom improvement |
| Lü J 2010 [71] | 14/11 | 15/10 | 46.8 | Not reported | Tap affected area with plum blossom needle followed by cupping on relevant acupoints once every 2 days, plus electroacupuncture for 30 minutes once daily | Electroacupuncture for 30 minutes once daily | 40 days | Cured, markedly effective, effective, ineffective based on symptom improvement |
| Ou X 2009 [75] | 60 (gender proportion not reported) | 48 (gender proportion not reported) | 48.6 | Medical textbook published in China: *Acupuncture Prescriptions for Common Diseases* | Flash cupping on relevant acupoints, herbal decoction twice daily, plusroutine body acupuncture for 30 minutes once daily | Herbal decoction twice daily, plus body acupuncture for 30 minutes once daily | 30 days | Cured, markedly effective, effective, ineffective based on symptom improvement |
| Qiu J 2003 [76] | 40 (gender proportion not reported) | 40 (gender proportion not reported) | Not reported | Not reported | Medicinal cupping (mixture of ginger, mustard, and dimethyl sulfoxide) on relevant acupoints for 30 minutes once daily, plus neurotrophic medication once daily (types and dosage not reported) | Neurotrophic medication once daily (types and dosage not reported) | 30 days | Cured, markedly effective, effective, ineffective based on symptom improvement |
| Ren Y 2006 [77] | 26/24 | 25/24 | Not reported | Medical textbook published in China: *2,000 Latest Domestic and Foreign Diagnostic Criteria* | Tap affected area with plum blossom needle, cupping on bilateral TB17 acupoints for 10 minutes once daily, plus routine body acupuncture for 30 minutes once daily | Acupuncture for 30 minutes once daily | 30 days | Cured, markedly effective, effective, ineffective based on symptom improvement |
| Sun H 2010 [80] | 28/12 | 25/11 | 44.6 | Not reported | Tap affected area with plum blossom needle , cupping once every two days on TB17acupoints for 10 minutes once every two days, plus routine body acupuncture for 30 minutes once daily | Routine body acupuncture for 30 minutes once daily | 15 days | Cured, markedly effective, effective, ineffective based on symptom improvement |
| Wang L 2010 [89] | 32/28 | 34/26 | Not reported | Medical textbook published in China: *2,000 Latest Domestic and Foreign Diagnostic Criteria* | Tap affected area with plum blossom needle, cupping on relevant acupoints until 3 ml to 5 ml of blood are let once every two days, plus routine acupuncture for 30 minutes once daily | Routine acupuncture for 30 minutes once daily | 30 days | Cured, markedly effective, effective, ineffective based on symptom improvement |
| Zhao N 2010 [133] | 27/16 | 25/17 | Not reported | Medical textbook published in China: *Neurology* | Flash cupping on relevant acupoints for 5 minutes, plus routine acupuncture for 30 minutes, once daily | Routine acupuncture for 30 minutes once daily | 30 days | Cured, markedly effective, effective, ineffective based on symptom improvement |
| Zhu F 2009 [141] | 20/14 | 18/16 | 33.1 | Not reported | Tap affected area with plum blossom needle, flash cupping on relevant acupoints for 3 minutes once daily after 7 days of the needling and cupping were applied, plus routine acupuncture once daily | Antiviral and neurotrophic medication (types and dosage not reported) each once daily , after 7 days of medications were started, plus acupuncture once daily | 30 days | Cured, markedly effective, effective, ineffective based on symptom improvement |

Definitions of “cure”, “markedly effective”, “effective”, and “ineffective”:

*Cured: All clinical symptoms resolved, no facial sensory disturbance.

Markedly effective: Clinical symptoms significantly alleviated; facial sensory disturbance is obviously effective.

Effective: Clinical symptoms alleviated, sensory disturbance somewhat effective.

Ineffective: No alleviation of clinical symptoms or of facial sensory disturbance.
