# Supplementary material for: An Updated Review of the Efficacy of Cupping Therapy
Source: PLoS One. 2012 Feb 28;7(2):e31793. doi: 10.1371/journal.pone.0031793 (PMC3289625; doi:10.1371/journal.pone.0031793)
Supplement: Table S4 — Characteristics of 6 included trials on cupping for acne. (DOC) [file pone.0031793.s004.doc]

**Table S4 Characteristics of 6 included trials on cupping for acne**

| **Trials** | **Patients (M/F)** | | **Average age (y)** | **Diagnostic**  **criteria** | **Interventions** | | **Duration of treatment** | **Outcome measure** |
| --- | --- | --- | --- | --- | --- | --- | --- | --- |
|  | **Treatment** | **Control** |  |  | **Cupping treatment** | **Control** |  |  |
| Huang J 2010 [38] | 76 (gender proportion not reported) | 50 (gender proportion not reported) | 23 | Medical textbook published in China: *Guiding Principles in Chinese Medicine* | Prick 5 back *shu* acupoints with tri-ensiform needle followed by cupping on same acupoints for 5 to 10 minutes twice weekly, plus herbal decoction 50 ml three times daily, and external facial cream | Herbal decoction 50 ml three times daily plus external facial cream | Not reported | *Cured, markedly effective, ineffective |
| Liu H 2009 [59] | 14/29 | 39 (gender proportion not reported) | 23.8 | Medical textbook published in China: *Guiding Principles in Chinese Medicine* | Flash cupping on lesions until face is flushed, plus routine acupuncture for 30 minutes once daily first 10 days, then once every 2 days for another 10 days | Routine acupuncture for 30 minutes once daily first 10 days, then once every 2 days for another 10 days | 30 days | *Cured, markedly effective, ineffective;  number of papules, pustules, cysts |
| Wang Q 2007 [91] | 30 (gender proportion not reported) | 30 (gender proportion not reported) | Not reported | Medical textbook published in China: *Acupuncture and Moxibustion Treatment* | Tap 5 back *shu* acupoints with plum blossomed needle followed by cupping on same acupoints for 5to 10 minutes once every 3 days, plus moving cupping on back and routine acupuncture for 30 minutes once daily | Routine acupuncture 30 minutes once daily | 30 days | *Cured, markedly effective, ineffective |
| Wu F 2010 [95] | 30 (gender proportion not reported) | 28 (gender proportion not reported) | Not reported | Not reported | Prick 2 back *shu* acupoints with tri-ensiform needle followed by cupping on same acupoints for 5 to 10 minutes once every 5 to 7 days | Tanshinone 1 g, three times daily | 30 days | *Cured, markedly effective, ineffective. |
| Wu Y 2008 [97] | 9/21 | 7/23 | 25.5 | Medical textbook published in China: *Dermatology* | Prick back *ashi* acupoints with tri-ensiform needle followed by cupping on same acupoints; cups are retained until 5to 7 ml blood is let; treat once daily | Tetracycline 0.25 g four times daily, plus 0.2% ketoconazole topical cream once daily | 10 days | *Cured, markedly effective, ineffective. |
| Zhang K 2008 [125] | 25/18 | 28/15 | Not reported | Not reported | Prick back *ashi*  acupoints with tri-ensiform needle followed by cupping on same acupoints for 10 to 15 minutes, once every two days | Tetracycline 500 mg, three times daily, plus external cream (name not reported) twice daily | 15 days | **Cured, markedly effective, ineffective. |

Definition of “cured”, “markedly effective”, “effective”, and “ineffective”:

Cured: Lesions completely or more than 95% cleared, clinical symptoms resolved, only mild pigmentation and scarring remain.

Markedly effective: Lesions cleared more than *70% (**60%), severity of lesions significantly alleviated.

Effective: Lesions cleared *30% to 69% (**20% to 59%), severity of lesions reduced.

Ineffective: Lesions cleared less than *30% (**20%), or lesions worsened.
