# Supplementary material for: An Updated Review of the Efficacy of Cupping Therapy
Source: PLoS One. 2012 Feb 28;7(2):e31793. doi: 10.1371/journal.pone.0031793 (PMC3289625; doi:10.1371/journal.pone.0031793)
Supplement: Table S5 — Characteristics of 6 included trials on cupping for cervical spondylosis. (DOC) [file pone.0031793.s005.doc]

**Table S5 Characteristics of 6 included trials on cupping for cervical spondylosis**

| **Trials** | **Patients (M/F)** | | **Average age (y)** | **Diagnostic**  **criteria** | **Interventions** | | **Duration of treatment** | **Outcome measure** |
| --- | --- | --- | --- | --- | --- | --- | --- | --- |
|  | Treatment | Control |  |  | **Cupping treatment** | **Control** |  |  |
| Shao M 2003 [79] | 40/53 | 41/48 | 47.5 | TCM practice guideline for diagnosis and defining treatment efficacy | Tap GV14 acupoint with plum blossom needle followed by cupping on the same acupoint for 8 to 10 minutes, plus routine acupuncture on bilateral EX-B2 acupoints for 20 minutes, twice weekly | Routine acupuncture on bilateral EX-B2 acupoints for 20 minutes, twice weekly | 35 days | *cure, markedly effective, ineffective |
| Wan XW 2007 [86] | 19/11 | 18/12 | 29.9 | Medical textbook published in China: *Guiding Principles in Chinese Medicine* | After routine acupuncture on bilateral GB20, bilateral EX-B2, and *ashi* acupoints needles retained for 10 minutes then removed, cupping was immediately applied over each needle hole using appropriate vacuum glass jar for 3 to 5 minutes, treatment was applied once daily | Routine acupuncture on bilateral GB20, bilateral EX-B2, and *ashi* points for 10 minutes, once daily | 30 days | *recovery, improvement, failure; treatment course; relapse rate |
| Wang PL 2010 [90] | 42 (gender proportion not reported) | 42 (gender proportion not reported) | 45.7 | TCM practice guideline for diagnosis and defining treatment efficacy | Tap GV14, bilateral EX-B2, bilateral GB21, bilateral SI11, and *ashi* acupoints with plum blossom needle followed by cupping on the same acupoints for 10 to 15 minutes once daily, plus routine acupuncture on abdominal acupoints for 30 minutes once daily | Routine acupuncture on abdominal acupoints for 30 minutes once daily | 30 days | *cure, markedly effective, effective, ineffective |
| Wang XM 2004 [93] | 29/37 | 13/17 | Not reported | Not reported | Prick GV14, bilateral BL11, bilateral EX-B2 with tri-ensiform needle followed by cupping on same acupoints for 10 to 15 minutes once every two days, plus electroacupuncture on the same acupoints during the same session | Electroacupuncture GV14, bilateral BL11, bilateral EX-B2 once every two days | 30 days | *cure, markedly effective, effective, ineffective. |
| You Y 2006 [116] | 18/12  (1 drop out) | 17/13 (2 drop outs) | 45.3 | Shenzhen Health Department practice guideline for TCM diagnosis and defining treatment efficacy | Tap acupoints on the Triple Burner and Small Intestine channels with plum blossom needle followed by cupping on the same acupoints for 5 minutes, plus 30 minutes traction once daily | 30 minutes traction once daily | 20 days | *cure, markedly effective, ineffective. |
| Zeng HW 2007 [117] | Group 1: 40 (gender proportion not reported) | Group 1: 40 (gender proportion not reported) | 47 | Criteria a established at national symposium on cervical spondylosis in 1992 | Group 1: Prick GV14 acupoint and bilateral C6 level acupoints with tri-ensiform needle followed by cupping onsame acupoints for 8 minutes, plus routine acupuncture and moxibustion on bilateral GB20 and EX-HN15 acupoints once daily | Group 1: Routine acupuncture and moxibustion on bilateral GB20 and EX-HN15 acupoints once daily | 20 days | ** markedly effective, ineffective; specific viscosity of blood; hemortheological parameters |
|  | Group 2: 40 (gender proportion not reported) | Group 2: 40 (gender proportion not reported) |  |  | Group 2: Prick GV14 acupoint and bilateral C6 level acupoints with tri-ensiform needle followed by cupping on the same acupoints for 8 minutes, once daily | Group 2: Flunarizine 5 mg once daily |  |  |

Definition of “cure”, “markedly effective”, “effective”, and “ineffective”:

Cured: Clinical symptoms resolved, the cervical or limb functions restored to normal.

Markedly effective: Clinical symptoms significantly alleviated, cervical and limb functions effective.

Effective: Clinical symptoms alleviated, but cervical or limb functions remain impaired.

Ineffective: Clinical symptoms and signs remain unchanged after the treatment.
