# Supplementary material for: An Updated Review of the Efficacy of Cupping Therapy
Source: PLoS One. 2012 Feb 28;7(2):e31793. doi: 10.1371/journal.pone.0031793 (PMC3289625; doi:10.1371/journal.pone.0031793)
Supplement: Table S6 — Characteristics of randomized controlled trials outside meta-analysis. (DOC) [file pone.0031793.s006.doc]

**Table S6: Characteristics of randomized controlled trials outside meta-analysis**

| **Type of intervention** | **Included disease** | **No. of trials** | **No. of**  **participants**  **(Treatment/Control)** | **Methodological quality** | **Main finding** |
| --- | --- | --- | --- | --- | --- |
| **Cupping versus no treatment** | Brachialgia paraesthetica nocturna | 1 | 10/10 | Not reported risk of bias | Wet cupping therapy is better than no treatment for symptom improvement based on visual analog scale scores |
| **Cupping versus usual care** | Wound and abscess | 4 | 370/339 | High risk of bias | Cupping therapy is significantly better than usual care for symptom improvement |
|  | Nonspecific low back pain | 3 | 110/108 | Not reported risk of bias | Cupping therapy is significantly better than usual care for pain relief |
|  | Symptoms of cough and dyspnea | 3 | 640/381 | High risk of bias | Cupping therapy is significantly better than usual care for symptom improvement |
|  | Acute lumbar muscle sprain | 3 | 108/106 | High risk of bias | Cupping therapy is significantly better than usual care for pain relief |
|  | Common cold | 2 | 130/80 | High risk of bias | Cupping therapy is significantly better than usual care for symptom improvement |
|  | Chronic obstructive pulmonary disease | 2 | 72/70 | High risk of bias | Cupping therapy is significantly better than usual care for symptom improvement |
|  | External humeral epicondylitis | 1 | 60/60 | Not reported risk of bias | ***No significant difference between cupping and usual care for symptom improvement*** |
|  | Lateral femoral cutaneous neuritis | 1 | 77/71 | High risk of bias | Cupping therapy is significantly better than usual care for symptom improvement |
|  | Cancer pain | 1 | 30/30 | High risk of bias | Cupping therapy is significantly better than usual care for pain relief |
|  | Stroke | 1 | 40/40 | High risk of bias | Cupping therapy is significantly better than usual care for symptom improvement |
|  | Insomnia | 1 | 20/20 | High risk of bias | Cupping therapy is significantly better than usual care for symptom improvement |
|  | Obesity | 1 | 42/33 | High risk of bias | Cupping is superior to usual care for decreasing of waist circumference and has similar effective on weight reduction |
|  | Carpal tunnel syndrome | 1 | 26/26 | Not reported risk of bias | Cupping therapy is significantly better than usual care for symptom improvement |
|  | Venomous snake bite | 1 | 50/50 | High risk of bias | Cupping therapy is significantly better than usual care for symptom improvement |
|  | Nausea/vomiting | 1 | 30/30 | High risk of bias | Cupping therapy is significantly better than usual care for symptom improvement |
|  | Osteoarthritis | 1 | 90/45 | High risk of bias | Cupping therapy is significantly better than usual care for symptom improvement |
|  | Upper-back myofasciitis | 1 | 45/45 | High risk of bias | Cupping therapy is significantly better than usual care for symptom improvement |
|  | Edema of the upper extremity after surgery | 1 | 34/33 | High risk of bias | Cupping therapy is significantly better than usual care for symptom improvement |
|  | Chronic gastritis | 1 | 56/56 | High risk of bias | Cupping therapy is significantly better than usual care for symptom improvement |
|  | Post-operative retention of urine | 1 | 40/40 | High risk of bias | Cupping therapy is significantly better than usual care for symptom improvement |
|  | Pain in low back and lower extremities | 1 | 46/41 | High risk of bias | Cupping therapy is significantly better than usual care for symptom improvement |
|  | Atherosclerosis | 1 | 22/20 | High risk of bias | Cupping therapy is significantly better than usual care for symptom improvement |
|  | Inflammation of superior cluneal nerves | 1 | 80/75 | High risk of bias | Cupping therapy is significantly better than usual care for symptom improvement |
|  | Prolapse of lumbar intervertebral disc | 1 | 60/60 | High risk of bias | Cupping therapy is significantly better than usual care for symptom improvement |
| **Combination of cupping and herbal medicine versus herbal medicine alone** | Herpes zoster | 1 | 56/56 | High risk of bias | Combination of cupping therapy and herbal medicine is significantly better than herbal medicine alone for symptom improvement |
|  | Acne | 1 | 76/50 | High risk of bias | Combination of cupping therapy and herbal medicine is significantly better than herbal medicine alone for symptom improvement |
|  | Leucoderma | 1 | 40/40 | High risk of bias | Combination of cupping therapy and herbal medicine is significantly better than herbal medicine alone for symptom improvement |
|  | Symptoms of cough and dyspnea | 1 | 30/28 | High risk of bias | Combination of cupping therapy and herbal medicine is significantly better than herbal medicine alone for symptom improvement |
|  | Perimenopausal syndrome | 1 | 30/30 | High risk of bias | Combination of cupping and herbal medicine is significantly better than herbal medicine alone for symptom improvement |
| **Combination of cupping, acupuncture and medication versus acupuncture and medications** | Herpes zoster | 1 | 40/40 | High risk of bias | Combination of cupping therapy and other treatment is significantly better than other treatment alone ofor symptom improvement |
|  | Facial paralysis (Bell palsy) | 1 | 60/48 | High risk of bias | Combination of cupping therapy and other treatment is significantly better than other treatment alone for symptom improvement |
| **Combination of cupping and acupuncture versus acupuncture alone** | Facial paralysis (Bell palsy) | 13 | 739/683 | High risk of bias | Combination of cupping and acupuncture is significantly better than acupuncture alone for symptom improvement |
|  | Cervical spondylosis | 5 | 231/182 | High risk of bias | Combination of cupping and acupuncture is significantly better than acupuncture alone for symptom improvement |
|  | Obesity | 4 | 132/125 | High risk of bias | ***Three studies showed cupping plus acupuncture had significant effect on reducing weight, one study showed no significant effect for combination treatment group compared with acupuncture alone*** |
|  | Herpes zoster | 3 | 101/92 | High risk of bias | Combination of cupping and acupuncture is significantly better than acupuncture alone for symptom improvement |
|  | Chloasma | 3 | 148/110 | High risk of bias | Combination of cupping and acupuncture is significantly better than acupuncture alone for symptom improvement |
|  | Prolapse of lumbar intervertebral disc | 3 | 125/125 | High risk of bias | Combination of cupping and acupuncture is significantly better than acupuncture alone for symptom improvement |
|  | Hand-shoulder syndrome | 3 | 95/95 | High risk of bias | Combination of cupping and acupuncture is significantly better than acupuncture alone for symptom improvement |
|  | Scapulohumeral periarthritis | 2 | 90/88 | High risk of bias | Combination of cupping and acupuncture is significantly better than acupuncture alone for symptom improvement |
|  | Acne | 2 | 73/69 | High risk of bias | Combination of cupping and acupuncture is significantly better than acupuncture alone for symptom improvement |
|  | Insomnia | 1 | 50/50 | High risk of bias | Combination of cupping and acupuncture is significantly better than acupuncture alone for symptom improvement |
|  | Facial spasm | 1 | 25/23 | High risk of bias | Combination of cupping and acupuncture is significantly better than acupuncture alone for symptom improvement |
|  | Osteoarthritis | 1 | 20/30 | High risk of bias | Combination of cupping and acupuncture is significantly better than acupuncture alone for symptom improvement |
|  | Acute ankle sprain | 1 | 46/46 | High risk of bias | Combination of cupping and acupuncture is significantly better than acupuncture alone for symptom improvement |
|  | Functional dyspepsia | 1 | 42/33 | High risk of bias | Combination of cupping and acupuncture is significantly better than acupuncture alone for symptom improvement |
|  | Chronic diarrhea | 1 | 30/30 | High risk of bias | Combination of cupping and acupuncture is significantly better than acupuncture alone for symptom improvement |
|  | Nonspecific subclinical disease | 1 | 32/30 | High risk of bias | Combination of cupping and acupuncture is significantly better than acupuncture alone for symptom improvement |
|  | Diabetic peripheral neuropathy | 1 | 33/32 | High risk of bias | Combination of cupping and acupuncture is significantly better than acupuncture alone for symptom improvement |
|  | Sciatica | 1 | 48/38 | High risk of bias | Combination of cupping and acupuncture is significantly better than acupuncture alone for symptom improvement |
|  | Knee pain | 1 | 34/38 | High risk of bias | Combination of cupping therapy and acupuncture is significantly better than acupuncture alone for pain relief |
|  | Nausea/vomiting | 1 | 16/16 | High risk of bias | Combination of cupping therapy and acupuncture is significantly better than acupuncture alone for symptom improvement |
|  | Depression | 1 | 36/36 | Not reported risk of bias | Combination of cupping and acupuncture is superior to acupuncture alone on improving depression symptoms based on Hamilton Rating Depression Scale |
| **Combination of cupping and medications versus medications alone** | Herpes zoster | 6 | 211/193 | High risk of bias | Combination of cupping and medication is significantly better than medication alone for symptom improvement |
|  | Symptoms of cough and asthma | 4 | 183/171 | High risk of bias | Combination of cupping and medication is significantly better than medication alone for symptom improvement |
|  | Facial paralysis (Bell palsy) | 3 | 106/106 | High risk of bias | Combination of cupping and medication is significantly better than medication alone for symptom improvement |
|  | Chronic obstructive pulmonary disease | 1 | 40/40 | High risk of bias | Combination of cupping and medication is significantly better than medication alone for symptom improvement |
|  | Intracranial hypertension | 1 | 40/40 | High risk of bias | Combination of cupping and medication is significantly better than medication alone for symptom improvement |
| **Combination of other TCM treatment versus other TCM treatment alone** | Prolapse of lumbar intervertebral disc | 2 | 61/60 | High risk of bias | Combination of cupping and traction is significantly better than traction alone for symptom improvement |
|  | Vertigo | 1 | 50/47 | High risk of bias | Combination of cupping and manual traction is significantly better than manual traction alone for symptom improvement |
|  | Cervical spondylosis | 1 | 30/30 | High risk of bias | Combination of cupping and traction is significantly better than traction alone for symptom improvement |
|  | Ulcerative colitis | 1 | 15/15 | High risk of bias | Combination of cupping and moxibustion is significantly better than moxibustion alone for symptom improvement |
