# Supplementary material for: An Updated Review of the Efficacy of Cupping Therapy
Source: PLoS One. 2012 Feb 28;7(2):e31793. doi: 10.1371/journal.pone.0031793 (PMC3289625; doi:10.1371/journal.pone.0031793)
Supplement: Protocol S1 — Flow chart of search strategy for inclusion and exclusion of studies. (DOC) [file pone.0031793.s008.doc]

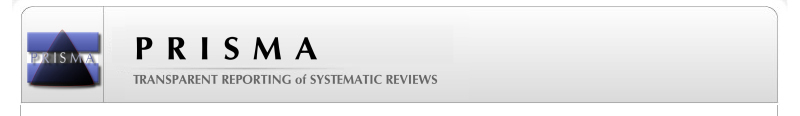
**PRISMA 2009 Flow Diagram**

**Screening**

**Included**

**Eligibility**

**Identification**

Records identified through 2008 after initial search of 6 databases n = 4,696

New records identified 2009 through 2010 after search of 6 databases n = 1,294

Records screened
n = 5,990

Records excluded
n = 5,332

Full-text articles assessed for eligibility
n =658

Full-text articles excluded due to non-randomized controlled trials, case reports, reviews and basic/mechanism studies

n = 523

Studies included in qualitative synthesis
n = 135

Studies included in quantitative synthesis
 (meta-analysis)
n = 42
